# Phosphorus-Doping Enables the Superior Durability of a Palladium Electrocatalyst towards Alkaline Oxygen Reduction Reactions

**DOI:** 10.3390/ma17122879

**Published:** 2024-06-12

**Authors:** Wen-Yuan Zhao, Miao-Ying Chen, Hao-Ran Wu, Wei-Dong Li, Bang-An Lu

**Affiliations:** 1 College of Materials Science and Engineering, Zhengzhou University, Zhengzhou 450001, China; zwy2533@163.com (W.-Y.Z.); chenmiaoying11@163.com (M.-Y.C.); whr1919566537@outlook.com (H.-R.W.); liweidong2023@gs.zzu.edu.cn (W.-D.L.); 2 International College, Zhengzhou University, Zhengzhou 450001, China

**Keywords:** oxygen reduction reaction, palladium, nonmetallic doping, phosphorization, durability

## Abstract

The sluggish kinetics of oxygen reduction reactions (ORRs) require considerable Pd in the cathode, hindering the widespread of alkaline fuel cells (AFCs). By alloying Pd with transition metals, the oxygen reduction reaction’s catalytic properties can be substantially enhanced. Nevertheless, the utilization of Pd-transition metal alloys in fuel cells is significantly constrained by their inadequate long-term durability due to the propensity of transition metals to leach. In this study, a nonmetallic doping strategy was devised and implemented to produce a Pd catalyst doped with P that exhibited exceptional durability towards ORRs. Pd_3_P_0.95_ with an average size of 6.41 nm was synthesized by the heat-treatment phosphorization of Pd nanoparticles followed by acid etching. After P-doping, the size of the Pd nanoparticles increased from 5.37 nm to 6.41 nm, and the initial mass activity (MA) of Pd_3_P_0.95_/NC reached 0.175 A mg_Pd_^−1^ at 0.9 V, slightly lower than that of Pd/C. However, after 40,000 cycles of accelerated durability testing, instead of decreasing, the MA of Pd_3_P_0.95_/NC increased by 6.3% while the MA loss of Pd/C was 38.3%. The durability was primarily ascribed to the electronic structure effect and the aggregation resistance of the Pd nanoparticles. This research also establishes a foundation for the development of Pd-based ORR catalysts and offers a direction for the future advancement of catalysts designed for practical applications in AFCs.

## 1. Introduction

As a renewable energy conversion device, fuel cells can effectively reduce the consumption of fossil fuels, especially in the transportation sector [1,2,3,4,5]. Alkaline fuel cells (AFCs) have garnered significant public interest owing to their expeditious electrochemical reaction kinetics and the extended operational lifetime resulting from their low-temperature operation [6,7,8,9]. Nevertheless, the sluggish kinetics of oxygen reduction reactions (ORRs) substantially hampers the energy efficiency of the battery and necessitates substantial amounts of scarce and costly Pt, thereby impeding their widespread implementation in fuel cells [10,11,12,13]. As an alternative to Pt-based catalysts, Pd-based catalysts have garnered increasing interest owing to their electronic structure similarity to platinum and comparatively greater mineral reserves [14,15,16,17,18]. As a result of its substandard durability and diminished intrinsic activity, it is critical to enhance the performance of Pd to fulfill the operational requirements of AFCs [19,20,21].

To achieve strict performance criteria, alloying, phase engineering, single-atom catalyst preparation, and particle size or morphology modification have been used [22,23,24,25,26]. Element doping may be the best way to develop enhanced electrochemical catalysts. The inevitable dissolution of base metals during the operation of fuel cells [27] and the produced metal cations result in membrane degradation by catalyzing Fenton reactions to produce aggressive *OH radicals [28,29]. Due to their unique features, nonmetal dopants have gained more attention than metallic element doping [30,31,32]. Nonmetals can potentially permeate platinum-group metal (PGM) interstitial sites due to their smaller radii, expanding their strain impact and electronic structural controls [33,34]. The nonmetal doping would produce substantial charge transfer and s, p-d hybridization with the host metals, unlike metal–metal alloys, which have d–d orbital coupling [30,34]. PGM catalysts doped with nonmetals may be durable due to the strong nonmetal–metal connection [35,36,37]. PGM-based catalysts are now doped with boron, nitrogen, sulfur, carbon, and hydrogen [14,30,38]. More importantly, the leaching of nonmetal elements produces anions, which will not catalyze the Fenton reaction and damage the membrane and ionomer [39].

Due to their corrosion resistance, metal phosphides are chemically stable in acidic and alkaline conditions [37]. Near-surface doping yielded a P_NS_-Pt/C catalyst with increased ORR activity and stability. After an accelerated durability test (ADT), the mass activity (MA) was 0.86 A mg_Pt_^−1^, and the mass activity loss was only 14%, far lower than commercial Pt/C (51%) [39]. Li et al. successfully synthesized PtP_2_ NPs through chemical vapor deposition (CVD), and its MA remained at 0.24 A mg_Pt_^−1^ even after 90,000 cycles of ADT [40]. In addition, due to the strong interaction between P and metals and the effective regulation of the electronic structure of active metal sites, palladium phosphide (Pd_x_P_y_) catalysts provide the possibility for constructing high-performance ORR electrocatalysts. Amorphous Pd-based nanoparticles have higher ORR activity and durability than crystalline ones. P-induced amorphization and metallic component expansion reduce free energy changes in the rate-determined step, explaining the positive association with catalytic activity [41].

Here, we report the successful synthesis of P-doped Pd nanoparticles (NPs) for ORRs using a simple CVD method. We used commercial carbon black (C, Vulcan XC-72R) and nitrogen-doped nanocarbon (NC) as support to load Pd particles, respectively. The experimental results showed that the P doping strategy effectively improved the ORR durability of the catalyst. Compared to the Pd/C catalyst, the Pd_3_P_0.95_/NC catalyst exhibited excellent catalytic stability in 0.1 M of a KOH electrolyte. The MA of Pd_3_P_0.95_/NC reached 0.175 A mg_Pd_^−1^ at 0.9 V, slightly lower than that of Pd/C. After 40,000 cycles of accelerated durability testing, the MA of Pd_3_P_0.95_/NC increased by 6.3%, while the MA loss of Pd/C was 38.3%.

## 2. Experimental Section

### 2.1. Chemicals and Reagents

Palladium chloride (PdCl_2_, Kunming Institute of Precious Metals, Kunming, China), Zinc nitrate hexahydrate (Zn(NO_3_)_2_ 6H_2_O, AR, Aldrich, Shanghai, China), 2-Methylimidazole (AR, Aldrich), Sodium hypophosphite (NaH_2_PO_2_ H_2_O, AR, Fuchen Tianjin Chemical Reagent Co., Ltd., Tianjin, China), Vulcan XC-72R carbon (Cabot, Shanghai, China), Sodium chloride (NaCl, AR, Sinopharm, Shanghai, China), Potassium hydroxide (KOH, AR, Sinopharm), Methanol (AR, Sinopharm), Ethanol (AR, Sinopharm), Ethylene glycol (EG, AR, Sinopharm), Hydrochloric acid (HCl, GR, 36%, Sinopharm), Perchloric acid (HClO_4_, GR, 70%, Sinopharm), Nafion solution (D520, 5 wt%, Alfa-Aesar, Shanghai, China), and Isopropyl alcohol (IPA, AR, Macklin, Shanghai, China) were used as received. All solutions were prepared with Ultrapure water (18.2 MΩ cm).

### 2.2. Materials Synthesis

Synthesis of Pd/C catalysts. In a 50 mL beaker, 20 mg of Vulcan XC-72R carbon with 10.0 mL of EG was added and then sonicated. Next, 100 μL of a Na_2_PdCl_4_ solution (53.2 mg_Pd_ mL^−1^) was added and stirred for 10 min. The solution was stirred while 10 mL of 5.6 mg mL^−1^ KOH/EG was added dropwise, and then further stirred for 10 min to ensure even mixing. Subsequently, the beaker was placed in the center of a microwave oven (20 W) with stirring. The reaction system temperature was raised to 130 °C and kept at this temperature for 180 s. After that, the system was cooled down to room temperature, and the pH value of the suspension was adjusted with the addition of 15 mL 0.1 M HCl for promoting Pd deposition onto carbon support. Next, the catalyst was separated from the resulting mixture by centrifuge twice after HCl pickling. The catalyst was dispersed with ethanol and sonicated for 2 h, filtered, washed with water, and dried in an oven at 60 °C for 12 h under vacuum to obtain Pd catalysts.

Synthesis of NC support. The nitrogen-doped nanocarbon (NC) support was derived from the pyrolysis of the Zeolitic Imidazolate Framework-8 (ZIF-8). In the standard procedure, 100 mL of a methanol solution was dissolved with 2.3 g of 2-Methylimidazole to yield a transparent, homogeneous solution denoted as solution A. Similarly, 2 g of Zn(NO_3_)_2_ 6H_2_O was dissolved in the same 100 mL of methanol solution to produce a uniform solution known as solution B. Twenty-four hours after combining solutions A and B, the reaction temperature was raised to 60 °C to promote the growth of ZIF-8 nanocrystals. After the ZIF-8 nanocrystals had cooled to room temperature, they were gathered via centrifugation, subjected to a minimum of three washes with methanol, and subsequently desiccated for 5 h at 60 °C in a vacuum oven. To obtain nitrogen-doped nanocarbons (NCs), the ZIF-8 precursors were subsequently heated in a tube furnace under Ar gas conditions for 1 h at 1100 °C [4,23].

Synthesis of Pd/NC catalysts. Briefly, Pd/NC catalyst samples were fabricated from a similar procedure to Pd/C, except for using NCs as the support.

Synthesis of Pd_3_P_0.95_/NC catalysts. Pd_3_P_0.95_/NC catalyst samples were synthesized by phosphating Pd/NC catalysts using NaH_2_PO_2_ H_2_O as the phosphorus source under an Ar atmosphere. In detail, 15 mg of Pd/NC and 30 mg of NaH_2_PO_2_ H_2_O were used in our experiment. The phosphating temperature was increased to 250 °C with a heating rate of 5 °C min^−1^ and then kept for 2 h under an Ar atmosphere. During the process, the flow rate of Ar was 20 sccm.

### 2.3. Physical Characterization

The transmission electron microscope (TEM) and elemental mapping were acquired using a JEM-F200 (JEOL, Akishima, Japan) with an electron acceleration voltage of 200 kV. X-ray diffraction (XRD) measurements were performed on an X′Pert PRO X-ray Diffractometer (PANalytical, Almelo, The Netherlands) using copper Kα radiation (λ = 1.5406 Å) at 40 kV, 40 mA. X-ray photoelectron spectroscopy (XPS) measurements were performed on an ESCA LAB 250 spectrometer (Thermo Fisher, Waltham, MA, USA) using Al Kα irradiation. The carbon peak at 284.8 eV was used as a reference to correct for charging effects. Raman measurements were performed on a Thermo DXR 2xi (Thermo Fisher, Waltham, MA, USA). The palladium concentrations were conducted on an Agilent 5110 ICP-OES (Agilent, Palo Alto, CA, USA).

### 2.4. Electrochemical Characterization

Electrode Preparation. The catalyst ink was prepared by sonicating a mixture of 2.0 mg of the catalyst, 660 μL of isopropanol, 330 μL of ultrapure water, and 10 μL of Nafion solution (5 wt%) for 30 min. The concentration of Pd was controlled to be 0.3 mg_Pd_ mL^−1^ based on the ICP-OES measurement. The catalyst ink (10 μL) was transferred onto the glassy carbon (GC) electrode and dried in air at room temperature.

Rotating disk electrode (RDE) tests. The electrochemical experiments were conducted on an electrochemical workstation (CHI 760E, SCHI, Shanghai, China) using a conventional three-electrode system, where the RDE that was connected to the installation of the rotating electrode speed control (Pine Research Instrumentation) was used as the working electrode. A graphite carbon rod and a saturated calomel electrode (SCE) were used as the counter and reference electrodes, respectively. All potentials reported in this paper were calibrated relative to the reversible hydrogen electrode (RHE). ORR measurements were conducted in O_2_-saturated 0.1 M KOH solutions at the scan rate of 10 mV s^−1^ from 0.1 to 1.2 V vs. the RHE with a rotation rate of 1600 rpm. In the ORR polarization curves, the current densities were normalized in reference to the geometric area of the GC RDE (0.196 cm^2^). Before the ORR measurement, the electrode was first scanned between 0.1 and 1.2 V at a scan rate of 100 mV s^−1^ in an N_2_-saturated 0.1 M KOH for a few cycles until it reached the steady state.

Rotating ring disk electrode (RRDE) tests. The H_2_O_2_ selectivity of the electrocatalyst was measured using the RRDE, and the potential of the Pt ring was set to 1.5 V vs. the RHE. The polarization curves were recorded at a scan rate of 10 mV s^−1^ from 0.1 to 1.2 V with a rotation rate of 1600 rpm at room temperature. The H_2_O_2_ selectivity (H_2_O_2_%) was calculated from the ring current (*I*_R_) and the disk current (*I*_D_) using the following equation:

(1) $$H_{2}O_{2}\%=\frac{200\times I_{R}}{\left(I_{R}+N\times I_{D}\right)}$$

where *N* = 0.37 is the current collection efficiency of the Pt ring.

The electron-transfer numbers were calculated using the following equation:

(2) $$n=\frac{4\times N\times I_{D}}{\left(I_{D}\times N+I_{R}\right)}$$

From the Koutecky–Levich (K–L) equation, we can obtain the kinetic current density *I*_K_.

(3) $$\frac{1}{I}=\frac{1}{I_{L}}+\frac{1}{I_{K}}$$

where *I* is the current density from practical measurements, and *I*_L_ and *I*_K_ are the diffusion-limited current density and the kinetic current density, respectively.

Catalyst Durability Test. The durability test was performed by cycling the catalyst between 0.6 and 1.0 V at 100 mV s^−1^ in O_2_-saturated 0.1 M KOH for 40,000 cycles. After every 10,000 cycles, the electrolyte was changed and purged with O_2_ for at least 20 min, and the polarization curve was recorded. The ORR performance of the catalysts was examined again between 0.1 to 1.2 V at 10 mV s^−1^ and 1600 rpm in fresh O_2_-saturated 0.1 M KOH.

## 3. Results and Discussion

**Figure 1:**
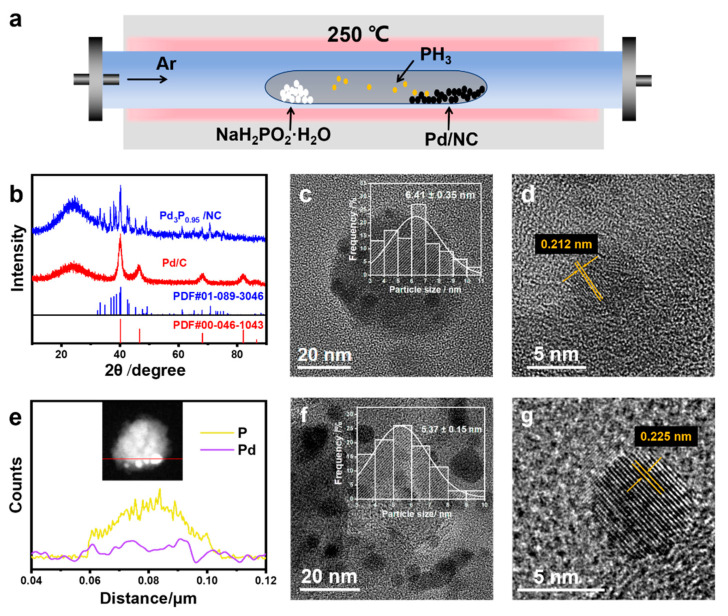
(**a**) Schematic illustration of the CVD method for the catalyst preparation, (**b**) XRD spectra of the catalysts, TEM images of (**c**) Pd_3_P_0.95_/NC and (**f**) Pd/C, Inset: particle size distribution statistical graph, HRTEM images of (**d**) Pd_3_P_0.95_/NC and (**g**) Pd/C, and (**e**) TEM image and corresponding elemental line scan result of Pd_3_P_0.95_/NC.

Briefly, the nitrogen-doped nanocarbon (NC) support was synthesized by the pyrolysis of ZIF-8 at 1100 °C under Ar. The Pd nanocrystals are obtained via a microwave-assisted glycol reduction method [42,43]. The final Pd_3_P_0.95_/NC catalysts were prepared using a CVD method on the pre-prepared Pd/NC (Figure 1a). In our study, sodium hypophosphite was used as the source of phosphorus, and no organic reagents were introduced, which met the requirements of fuel cell applications. In contrast, organic phosphine reagents, such as tri-n-octylphosphine, are considered toxic and expensive. The phase composition of Pd_3_P_0.95_/NC was initially studied using X-ray diffraction (XRD) (Figure 1b). The XRD pattern of Pd_3_P_0.95_/NC was consistent with the standard card of the Pd_3_P_0.95_ crystal (PDF No. 01-089-3046). The peaks at 33.2, 34.8, 36.9, 37.9, 38.7, 39.9, 40.3, 42.6, 43.1, 45.3, 49.2, and 70.9° were assigned to the (121), (201), (211), (102), (220), (112), (031), (221), (131), (122), (301), and (332) planes, corresponding to the Pd_3_P_0.95_ crystal structure based on the data of the standard PDF file (PDF #01-089-3046). By comparison, the Pd NPs synthesized without the introduction of P displayed a characteristic face-centered cubic (fcc) structure (PDF No. 00-046-1043) in the XRD pattern (Figure 1b). The peaks at 40.1, 46.7, 68.1, 82.1, and 86.6° were assigned to the (111), (200), (220), (311), and (222) planes, corresponding to the Pd crystal structure (PDF #00-046-1043). Figure S4 illustrates the XRD pattern of Pd/NC, which was comparable in nature to that of Pd/C. The morphological features of Pd_3_P_0.95_/NC were studied by transmission electron microscopy (TEM). As shown in Figure 1c, the Pd_3_P_0.95_ nanoparticles were uniformly anchored on NC support, with a narrow size distribution centered at ~6.41 nm of Pd_3_P_0.95_/NC. Figure 1d further reveals that the regular shape of Pd_3_P_0.95_ NPs and well-resolved lattice spacing of ~0.212 nm is observed, which is consistent with the lattice spacing of the Pd_3_P_0.95_ (221) crystal plane (Figure S6). The elemental line scan results (Figure 1e) showed the simultaneous existence of Pd and P elements in metal particles. The EDS elemental mappings demonstrated that Pd and P were evenly distributed throughout the entire nanoparticle of Pd_3_P_0.95_ (Figure S2). The same uniform dispersion of Pd particles in the Pd/C catalyst was demonstrated by the EDS mapping results (Figure S1). As a control, the average particle size of Pd NPs in Pd/NC synthesized by the same method was about 5.37 nm (Figure 1f), and the lattice spacing of the corresponding (111) crystal plane was ~0.225 nm (Figure 1g). It should be noted that slight a particle agglomeration of the particles was observed during the phosphating process, similar to previous results of PtP_2_ [40].

**Figure 2:**
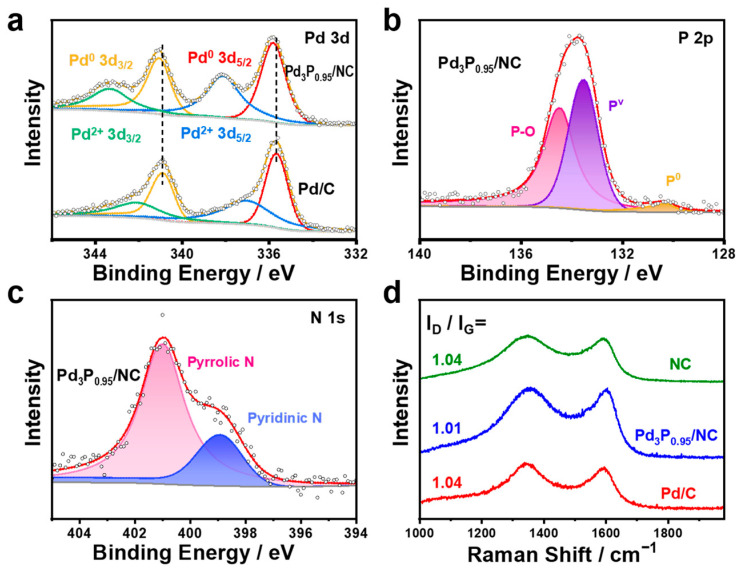
(**a**) Pd 3d XPS spectra of Pd_3_P_0.95_/NC and Pd/C, (**b**) P 2p XPS spectrum of Pd_3_P_0.95_/NC, (**c**) N 1s XPS spectrum of Pd_3_P_0.95_/NC, and (**d**) Raman spectra of NC, Pd_3_P_0.95_/NC, and Pd/C.

X-ray photoelectron spectroscopy (XPS) was performed to characterize the chemical state of the investigated electrocatalysts. We first analyzed the surface valence states of Pd_3_P_0.95_/NC and Pd/C to observe the effect of phosphorus-doping (Figure 2a). The 3d_3/2_ and 3d_5/2_ peaks of Pd^0^ showed a 0.15 eV positive shift compared to Pd/C (Table S1), which could be attributed to the electron migration from Pd to P [41,43]. Meanwhile, the P 2p XPS spectra of Pd_3_P_0.95_/NC are shown in Figure 2b, where the peaks at 130.3, 133.6, and 134.5 eV can be attributed to the elemental phosphorus (P^0^), phosphorus oxide (P^V^), and P-O species formed on the surface of the Pd_3_P_0.95_/NC [44]. The presence of P^0^ confirmed the successful introduction of P into the lattice of Pd. An electron density shift from Pd to P created electron vacancies in Pd and increased the probability of an electron transition from 2p to the unoccupied 5d orbital [37,41]. The N 1s fine spectra showed two peaks at 398.9 eV and 401.0 eV for pyridinic N and pyrrolic N in the Pd_3_P_0.95_/NC electrocatalyst (Figure 2c), similar to carbon nanomaterials derived from ZIF-8 reported by the previous literature [23]. Raman spectroscopy showed the obvious carbon structural information of disordered carbon (D, 1355 cm^−1^) and graphitic carbon (G, 1598 cm^−1^) bands. The I_D_/I_G_ ratios for NC, Pd_3_P_0.95_/NC, and Pd/C were 1.04, 1.01, and 1.04, respectively, indicating a high degree of graphitization of carbon support.

**Figure 3:**
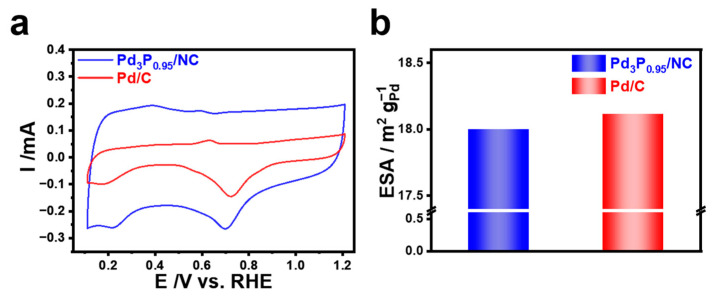
(**a**) CV curves of Pd_3_P_0.95_/NC and Pd/C recorded in 0.1 M KOH at a scan rate 100 mV s^−1^, and (**b**) histogram of calculated ESAs for investigated electrocatalysts.

Based on the cyclic voltammograms (CVs) recorded in the N_2_-saturated electrolyte, the electrochemically active surface area (ECSA) of the electrocatalysts was calculated by measuring the charge associated with the reduction of oxygen adsorption on Pd. The charge required to deposit hydrogen at the underpotential (UPD) is often utilized in the computation of the electrochemical active surface area (ECSA) for Pt. However, the accurate determination of the charge of the hydrogen UPD for Pd was not possible due to the interference caused by hydrogen absorption in the Pd lattice. We utilized the reduction charge of surface Pd(OH)_2_ in order to calculate the electrochemical specific area (ESA) (Figure S7). A total of 430 μC cm^−2^ was selected to represent the formation of an entirely covered Pd(OH)_2_ layer. The CV profiles of the two electrocatalysts, which were evaluated in N_2_-saturated 0.1 M KOH, are depicted in Figure 3a. The reduction peak between 0.7 and 0.8 V can be attributed to the reduction of Pd(OH)_2_. The 1.2 V potential limit was selected due to its correspondence with the Pourbaix pH-potential diagram, which specifies that this value is the maximum allowable voltage for the generation of Pd(OH)_2_ [45]. Moreover, the electrochemical active surface area of different catalysts is shown in Figure 3b. Pd_3_P_0.95_/NC exhibited a slightly smaller ESA of 18.00 m^2^ g^−1^, which was close to the value of Pd/C (18.12 m^2^ g^−1^) (Table S2). The smaller ESA of Pd_3_P_0.95_/NC mainly resulted from the particle growth during the introduction of P at high temperatures. In our work, the particle size of Pd increased by ~20% after P-doping. The incorporation of nonmetal atoms into PGMs is typically necessitated by elevated temperature, owing to the strong interatomic bonds among metal atoms. This process invariably results in particle growth and a substantial reduction in the quantity of active sites. Furthermore, it should be noted that Pd_3_P_0.95_/NC delivered a much higher double-layered current because the NC support had a higher area.

**Figure 4:**
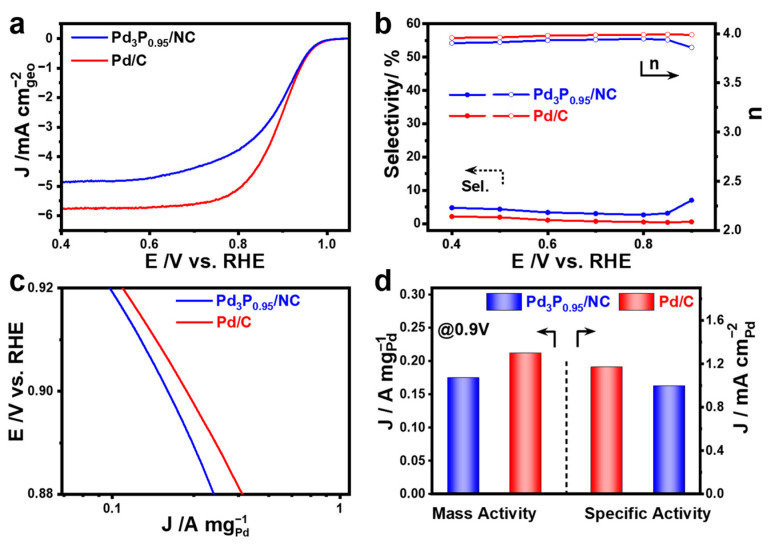
(**a**) ORR polarization curves, (**b**) values of the selectivity (%) and number of transferred electrons (*n*) calculated from the RRDE polarization curves, (**c**) mass activity Tafel plots of Pd_3_P_0.95_/NC and Pd/C, and (**d**) mass activity and specific activity at 0.9 V.

To evaluate the intrinsic activity of different electrocatalysts, we used the rotating disk electrode method and calculated the kinetic current density, *j*_k_, using the Koutecký–Levich equation. Figure 4a shows the ORR polarization curves of the investigated electrocatalysts tested in an O_2_-saturated 0.1 M KOH solution. Specifically, the polarization curve of Pd_3_P_0.95_/NC overlapped with that of Pd/C at a high potential above 0.9 V, indicating that Pd_3_P_0.95_/NC had similar ORR activity to Pd/C. Close to Pd/C, the half-wave potential of Pd/NC was 0.89 V, as illustrated in Figure S5. As a benchmark, Pd/C was utilized in our research. At the same time, subsequent experiments could confirm that after the stable CV cycles, the further activation of Pd_3_P_0.95_/NC resulted in a half-wave potential that was more positive than that of the initial one, indicating the excellent ORR activity of Pd_3_P_0.95_/NC (Figure S8). The rotating ring disk electrode (RRDE) method revealed that Pd_3_P_0.95_/NC and Pd/C mainly followed the 4e^−^ pathway during the ORRs (Figure 4b). By comparing the mass activity Tafel plots of the different catalysts, it was also proven that Pd_3_P_0.95_/NC had superior ORR activity (Figure 4c). The Tafel slope of Pd_3_P_0.95_/NC was 74.23 mV dec^−1^, while the value of Pd/C was 76.30 mV dec^−1^. The kinetic currents were normalized to the loading amount of Pd and ESA of each catalyst to calculate the mass and specific activity (Table S3). The actual Pd loading of different catalysts was determined according to the ICP results (Table S2). As shown in Figure 4d, the mass activity of Pd_3_P_0.95_/NC was slightly lower, 0.175 A mg^−1^, which was 0.826 times that of Pd/C (0.212 A mg^−1^). Meanwhile, the specific activity of Pd_3_P_0.95_/NC (0.998 mA cm^−2^) was 0.852 times that of Pd/C (1.171 mA cm^−2^) (Figure 4d and Table S3). Since the number of Pd sites on the surface was diminished by 15%, we hypothesized that the decreased initial activity could be attributed to the increased size following phosphorization.

**Figure 5:**
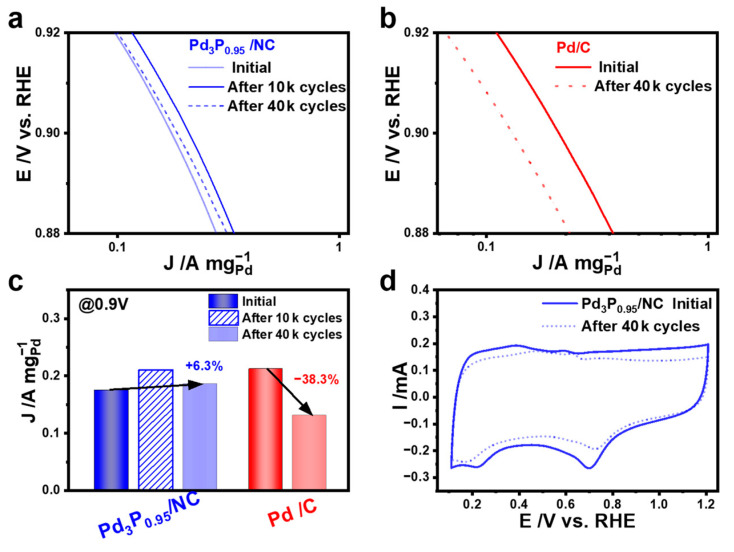
Mass activity Tafel plots before and after ADTs: (**a**) Pd_3_P_0.95_/NC; (**b**) Pd/C; (**c**) histograms of the mass activities of Pd_3_P_0.95_/NC and Pd/C. (**d**) CV curves of Pd_3_P_0.95_/NC before and after ADTs.

The long-term stability of electrocatalysts is one of the key standards for evaluating ORR performance [36,46]. The ORR durability of the investigated electrocatalysts was measured via accelerated durability tests (ADTs). The durability test was performed by cycling the catalyst between 0.6 and 1.0 V at 100 mV s^−1^ in O_2_-saturated 0.1 M KOH. The changes in the mass activity Tafel plots for Pd_3_P_0.95_/NC and Pd/C before and after stability tests prove that Pd_3_P_0.95_/NC had enhanced activity and stability. The decays of mass activity for different catalysts after ORR durability (Table S3) are summarized in Figure 5c, where the mass activity loss of Pd_3_P_0.95_/NC was much smaller than that of Pd. Figure 5a,b shows the mass activity Tafel plots of different catalysts before and after stability tests. Surprisingly, instead of decreasing, the performance of Pd_3_P_0.95_/NC increased from 0.175 A mg^−1^ to 0.210 A mg^−1^ after 10,000 CV cycles, which may be related to the full activation of the catalyst. Furthermore, compared to the initial activity, the ORR performance of Pd_3_P_0.95_/NC increased by 6.3% (0.186 A mg^−1^) even after the 40,000 cycles stability test (Figure 5a). In contrast, the remarkable loss of ORR activity of Pd/C was observed after the 40,000 cycle stability tests. As depicted in Figure 5b, the MA of Pd/C was 0.131 A mg^−1^ after ADT and 38.3% of activity was lost. Moreover, the CV curves of Pd_3_P_0.95_/NC before and after ADT revealed the loss of ECSA after ADT. The ECSA of Pd_3_P_0.95_/NC was reduced by 36.7% after ADT (Figure 5d). In contrast, the reduction peak of Pd(OH)_2_ in Pd/C was significantly reduced (Figure S3), and 52.9% of the active sites were diminished after ADT. These results demonstrate that the introduction of phosphorus can provide more aggregation resistance of Pd nanoparticles during ADT.

A comparison was made between the ORR catalytic performance and durability of the Pd_3_P_0.95_/NC catalyst and those documented in prior research (Table 1). Initial discussion focuses on palladium–nonmetal catalysts [30,38,41,47,48,49,50]. Additionally, as a control, studies of Pd-based metal alloys are added to the discussion [51,52,53]. The stability of palladium-based catalysts for alkaline ORR is evidently enhanced universally and significantly through nonmetal modification, such as S, Se, P, H, B, and other elements. From an electronegativity standpoint, the strong electronegativity of the elements in the main groups V A and VI A enables them to regulate the electronic structure of Pd effectively. On the other hand, the lattice gap of Pd can be traversed by B and H due to their diminutive atomic radius, which induces strain [38,49]. The adsorption of intermediates and the catalytic properties of the ORR can be influenced by alterations in the electronic structure induced by lattice expansion strain [24]. Yu et al. explored the active and stable Pd–Se alloy electrocatalysts with controlled phases toward alkaline ORRs [47]. The impact of crystal structures on the efficacy of oxygen reduction was elucidated through the comparison of Pd–Se catalysts exhibiting distinct crystal phases. With a mass activity of 0.460 A mg_Pd_^−1^ at 0.9 V versus RHE, Pd_17_Se_15_ NPs/C exhibited the best ORR performance. The Pd_7_Se_4_ NPs/C exhibited a mass activity of 0.186 A mg_Pd_^−1^. As a result of the more coordinated Se atoms, they discovered that the valence state of Pd in Pd_17_Se_15_ NPs/C was greater than that of Pd_7_Se_4_ NPs/C. After calculations and characterization, they concluded that Pd_17_Se_15_ provided more electrons to the *OOH intermediate in the potential determination step, which can account for the enhancement of the ORR activity. Another study came to similar conclusions about boosting ORRs. Yu et al. demonstrated a generalizable route to fabricate amorphous Pd-based nanomaterials by introducing phosphorus [41]. They concluded that P-induced amorphization can decrease the free energy changes in the rate-determined step (RDS). Du et al. [30] demonstrated that the monodispersion of the Pd_4_S leads to long-term stability and that the sites on the Pd_4_S surface could trap atomic oxygen moderately and desorb O_2_ facilely leading to high activity. As shown in Table 1, metal alloys can also regulate the electronic structure, but the stability of such catalysts is less than that of nonmetallic-doped catalysts. Through the data in Table 1, we believe that palladium-nonmetal catalysts have better stability under alkaline conditions. In our research, after 40,000 potential cycles between 0.6 V and 1.0 V, the MA of Pd_3_P_0.95_/NC paradoxically increased by 6.3%. Other previously reported catalysts showed varying degrees of decay after shorter stability test times. Compared with the stability results reported in similar literature, we believe that our catalyst has relatively good stability.

## 4. Conclusions

In summary, we provide a simple method for Pd-P nanocatalysts towards ORRs. Compared with Pd/C, Pd_3_P_0.95_/NC showed remarkable durability of ORRs in alkaline media. After 40,000 potential cycles between 0.6 V and 1.0 V, the MA of Pd_3_P_0.95_/NC paradoxically increased by 6.3% while the MA loss of Pd/C was 38.3%. Combining the experimental data, the enhanced durability was primarily ascribed to the electronic structure effect and the aggregation resistance of the Pd–P alloy. It is believed that the simple phosphating method presented in this work can provide a new way to design efficient Pd-based catalysts for AFCs.

## Figures and Tables

**Table 1:** The comparisons of the alkaline ORR performance of Pd_3_P_0.95_/NC and palladium-based alloy catalysts reported by other studies.

Catalyst	Initial-MA (mA µg_Pd_^−1^)	After ADT-MA (mA µg_Pd_^−1^)	Percentage Change (%)	Reference
Pd_3_P_0.95_/NC	0.175 (@ 0.90 V)	0.186 (40 k cycles)	+6.3	This work
Pd_17_Se_15_ NPs/C	0.460 (@ 0.90 V)	0.330 (15 k cycles)	−28.2	[47]
Pd_7_Se_4_ NPs/C	0.186 (@ 0.90 V)	0.123 (15 k cycles)	−33.9	
Pd-Te HPs/C	0.300 (@ 0.90 V)	0.252 (20 k cycles)	−16.0	[48]
Pd_4_S/C	0.130 (@ 0.85 V)	NA *	NA	[30]
B-Pd/C	0.970 (@ 0.90 V)	0.388 (3 k cycles)	−60.0	[49]
Pd_3_P@NPC	1.112 (@ 0.85 V)	0.951 (40 k cycles)	−14.5	[50]
Pd-H icosahedra/C	0.550 (@ 0.90 V)	0.503 (30 k cycles)	−8.5	[38]
P-Pd NPs/C	0.481 (@ 0.90 V)	0.405 (10 k cycles)	−15.9	[41]
PtPd NSs	0.382 (@ 0.80 V)	NA	NA	[51]
Pd_6_Ni/C	0.22 (@ 0.90 V)	0.208 (10 k cycles)	−5.6	[52]
PdCuCo NPs/C	0.13 (@ 0.90 V)	0.125 (10 k cycles)	−3.9	[53]

* NA (Not Applicable) indicates that this data is not provided in the literature.

## Data Availability

Data are contained within the article.

## Supplementary Materials

The following supporting information can be downloaded at: https://www.mdpi.com/article/10.3390/ma17122879/s1. Figures S1–S8: HAADF images and relevant EDS elemental mappings; CV curves; XRD spectra; LSV plots; schematic diagram of the reduction peak position; Tables S1–S3: XPS peak position; ICP-OES results; and ESA, SA, and MA.
