# Supplementary material for: Phosphorus-Doping Enables the Superior Durability of a Palladium Electrocatalyst towards Alkaline Oxygen Reduction Reactions
Source: Materials (Basel). 2024 Jun 12;17(12):2879. doi: 10.3390/ma17122879 (PMC11204636; doi:10.3390/ma17122879)
Supplement: Supplementary file 1 [file materials-17-02879-s001.zip › materials-3039086-supplementary.pdf]

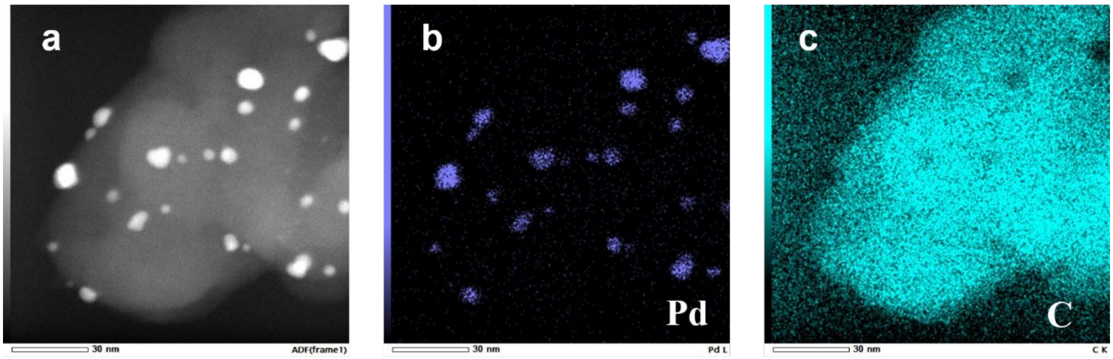

Figure S1. (a) HAADF image of Pd/C and relevant EDS elemental (Pd and C) mappings (b and c).

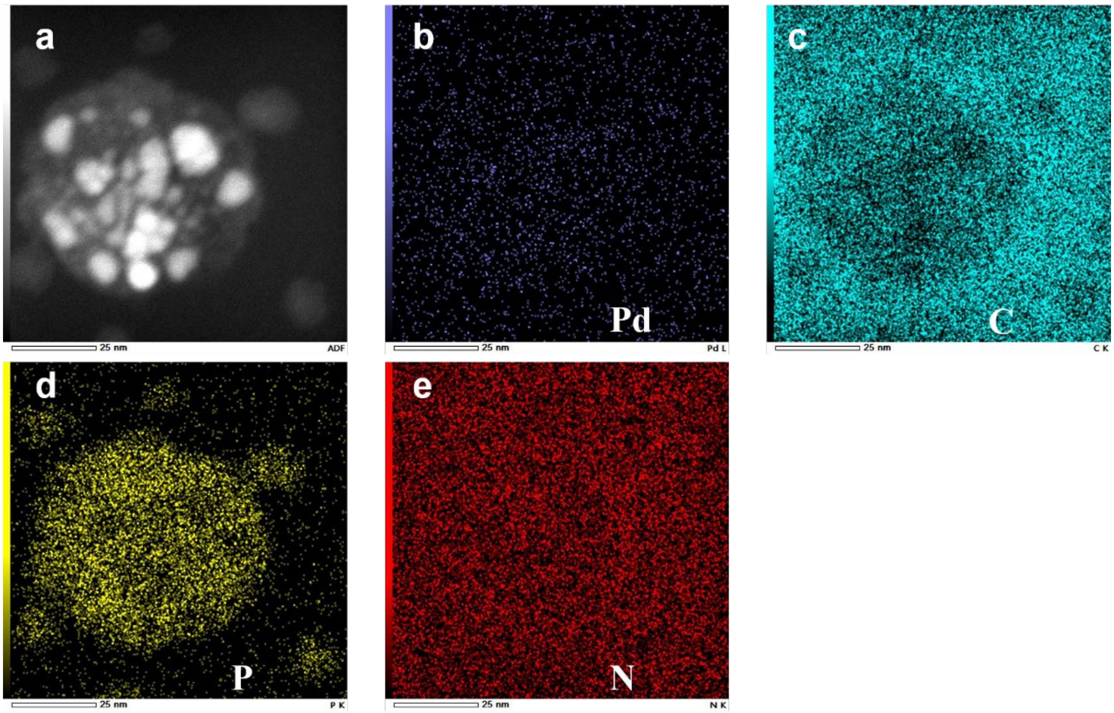

Figure S2. (a) HAADF image of $Pd_3P_{0.95}$/NC and relevant EDS elemental (Pd, C, P and N) mappings (b, c, d and e).

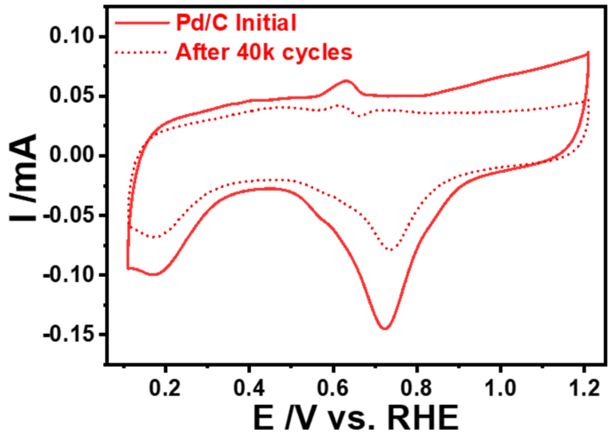

Figure S3. CV curves of Pd/C before and after ADT.

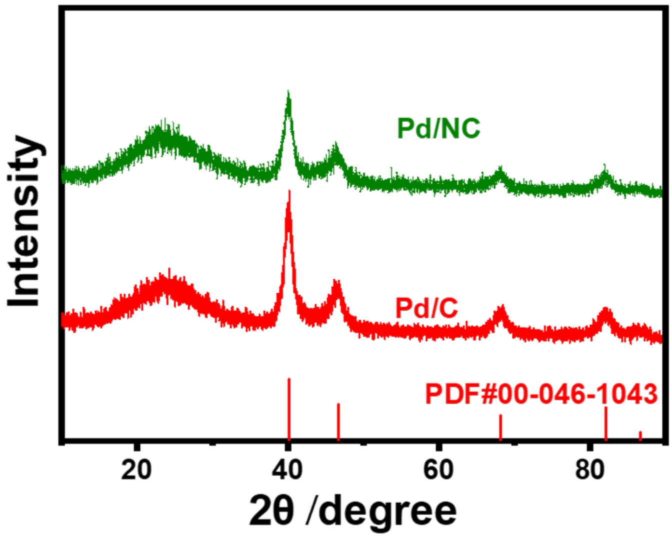

Figure S4. XRD spectra of Pd/NC and Pd/C.

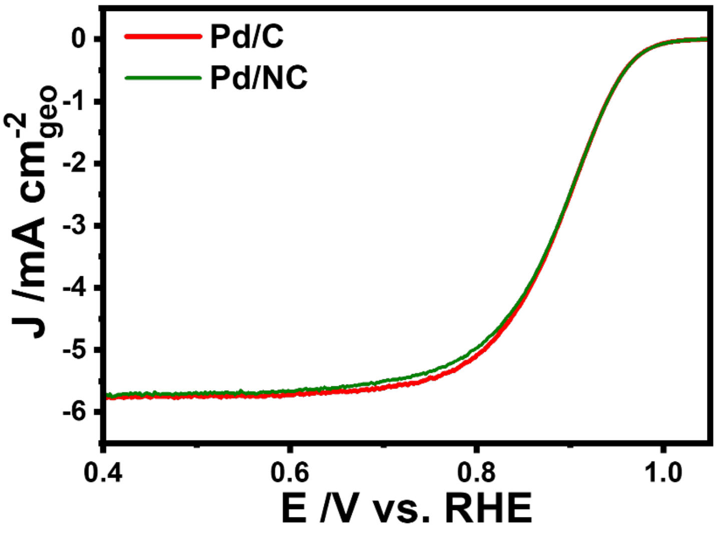

Figure S5. ORR polarization curves of Pd/NC and Pd/C.

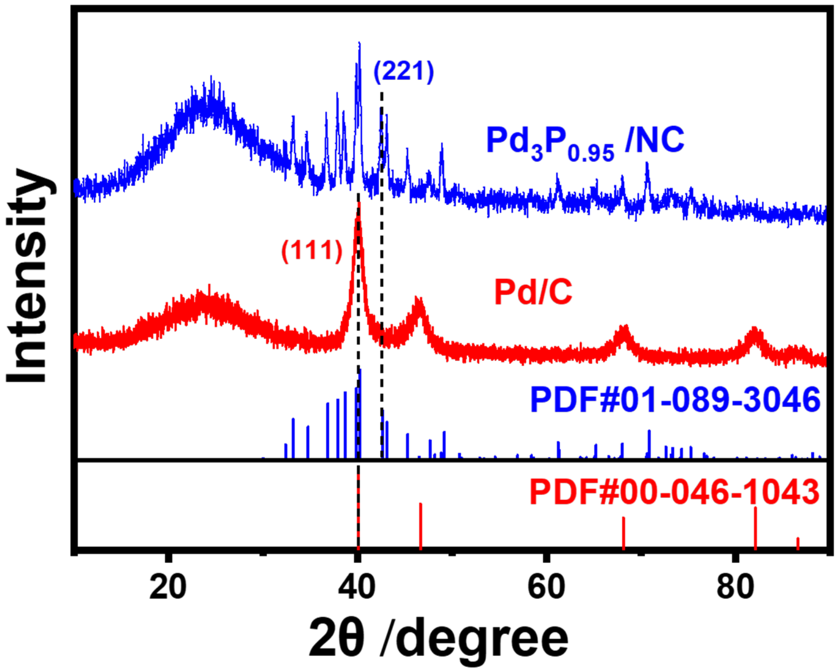

Figure S6. XRD spectra of catalysts and the marking of diffraction peaks corresponding to specific crystal faces displayed by TEM.

We used software to calculate the reduction charge of surface $Pd(OH)_2$. Using CHI760E software, select the last circle of the CV curve, select **Peak Definition** function, select **Gaussian** peak shape and determine. Select the **Manual Results** function, from the base of the peak to make the tangent line (**Figure S7**), can directly read the corresponding charge quantity value.

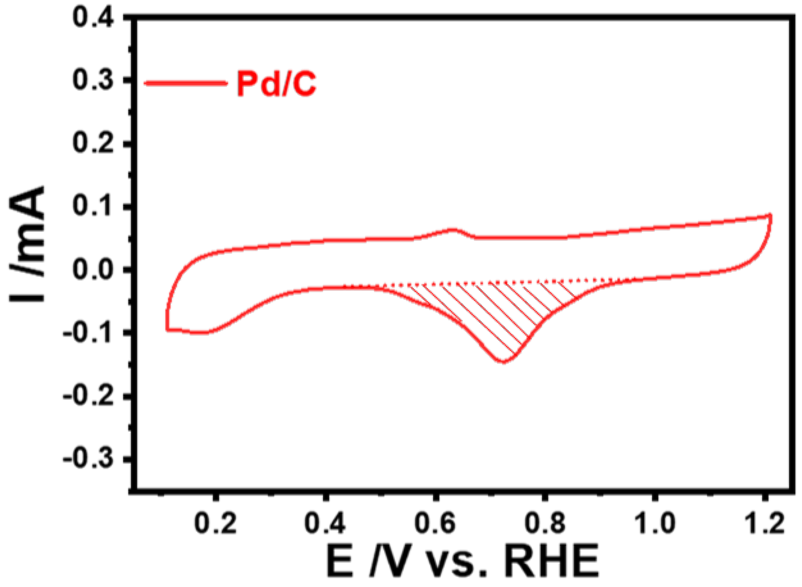

Figure S7. Schematic diagram of the reduction peak position of $Pd(OH)_2$.

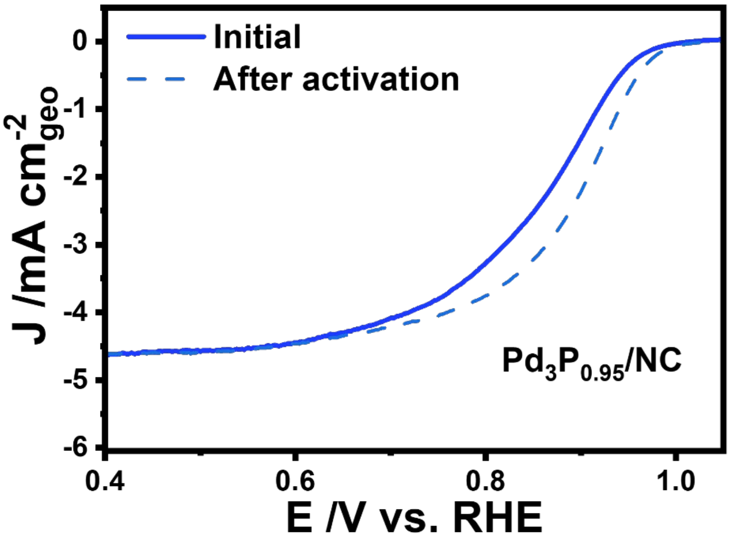

Figure S8. ORR polarization curves of $Pd_3P_{0.95}$/NC before and after activation.

Table S1. XPS peak position of Pd $3d_{5/2}$ for these two catalysts Pd/C and $Pd_3P_{0.95}$/NC.

Sample	Pd^0 $3d_{5/2}$ Position	Pd^0 $3d_{3/2}$ Position	Pd^{2+} $3d_{5/2}$ Position	Pd^{2+} $3d_{3/2}$ Position
Pd/C	335.67eV	341.05 eV	337.04 eV	342.20 eV
$Pd_3P_{0.95}$/NC	335.82eV	340.90 eV	338.12 eV	343.35 eV

Table S2. ICP-OES results of the prepared samples.

Sample	Pd (wt%)	P (wt%)
Pd/C	15.1455	-
$Pd_3P_{0.95}$/NC	15.5143	9.4953

Table S3. ESA, SA, and MA of catalysts before and after ADT.

Sample	Electrochemical specific area (m^2g^{-1})	Initial-SA, @0.90V (mA cm^{-2})	Initial-MA, @0.90V (mA μg_{Pd}^{-1})	10k-MA, @0.90V (mA μg_{Pd}^{-1})	40k-MA, @0.90V (mA μg_{Pd}^{-1})
Pd/C	18.12	1.171	0.212	0.180	0.131
$Pd_3P_{0.95}$/NC	18.00	0.998	0.175	0.210	0.186
